# Obesity as an effect modifier of the association between menstrual abnormalities and hypertension in young adult women: Results from Project ELEFANT

**DOI:** 10.1371/journal.pone.0207929

**Published:** 2018-11-28

**Authors:** Hui Xu, Peng-hui Li, Timothy M. Barrow, Elena Colicino, Changping Li, Ruixue Song, Hongbin Liu, Nai-jun Tang, Songyan Liu, Liqiong Guo, Hyang-Min Byun

**Affiliations:** 1 Department of Occupational & Environmental Health, School of Public Health, Tianjin Medical University, Tianjin, China; 2 Department of Epidemiology and Statistics, School of Public Health, Tianjin Medical University, Tianjin, China; 3 School of Environmental Science and Safety Engineering, Tianjin University of Technology, Tianjin, China; 4 Faculty of Health Sciences and Wellbeing, University of Sunderland, Sunderland, United Kingdom; 5 Icahn School of Medicine at Mount Sinai, New York, New York, United States of America; 6 Tianjin Research Institute for Family Planning, Tianjin, China; 7 School of Materials Science and Engineering, Chang'an University, Xi'an, Shanxi, China; 8 Human Nutrition Research Centre, Institute of Cellular Medicine, Newcastle University, Newcastle upon Tyne, United Kingdom; Michigan State University, UNITED STATES

## Abstract

**Background:**

The menstrual cycle is regulated by reproductive hormones such as estrogen which has been implicated in the pathogenesis of hypertension and is associated with obesity. However, to date there has scant study of hypertension in relation to menstrual characteristics and abnormalities. We hypothesize that adverse menstrual characteristics are associated with an increase the prevalence of hypertension and that this relationship is exacerbated by obesity.

**Methods:**

Our study leverages 178,205 healthy female participants (mean age = 29) in a population-based cross-sectional study in Tianjin, China. Menstrual characteristics including menstrual cycle length and regularity, menstrual bleeding length, menstrual blood loss and dysmenorrhea were assessed by self-reported questionnaires, and hypertension was diagnosed by physician. Multiple logistic regression models were used to assess the relationships between menstrual characteristics and hypertension.

**Results:**

Normal length menstrual cycle (OR = 1.21, 95% CI: 1.03–1.41), oligomenorrhea (OR = 1.54, 95% CI: 1.12–2.07), irregular cycle (OR = 1.54, 95% CI: 1.22–1.93), and light menstrual blood loss (OR = 1.36, 95% CI: 1.06–1.72) were associated with hypertension among women who are overweight or obese, but not among women who are normal weight. Longer menstrual bleeding duration (OR = 1.44, 95% CI: 1.24–1.67) and dysmenorrhea were associated with increased prevalence of hypertension (OR = 1.20, 95% CI: 1.14–1.41) in all young women.

**Conclusions:**

The prevalence of hypertension is higher among women with menstrual abnormalities, and this association is modified by overweight and obesity.

## Introduction

The menstrual cycle is regulated by many different reproductive hormones that are released by the hypothalamus and the pituitary gland [1]. Menstrual abnormalities include painful cramps (dysmenorrhea), heavy bleeding (menorrhagia), absence of menstruation (amenorrhea), light or infrequent menstruation (oligomenorrhea) and premenstrual syndrome (PMS)[2–8]. These can be caused by a range of factors, including hormonal imbalances, genetic factors, infection, stress, and clotting disorders [9, 10], and are associated with complications such as anemia, osteoporosis, and in severe cases, infertility [11]. Abnormal menstrual patterns caused by altered hormonal balance may mediate the risk of hypertension. For example, estrogen can play a role in protecting against cardiovascular diseases (CVDs) including hypertension by vasodilator function [12, 13], while androgens may increase blood pressure and thereby contribute to the pathogenesis of hypertension [14, 15]. Furthermore, many of the known risk factors for menstrual abnormalities, such as weight, age, smoking, family history, pregnancy history and stress [16], are also risk factors for the development of hypertension. However, to date there are few studies that have reported the prevalence of hypertension in women with menstrual abnormalities.

Obesity is associated with increased risk of hypertension, accounting for more than two-thirds of essential hypertension [17]. More than one-third of adults in the world are overweight or obese, and therefore it represents a threat to global health [18]. Furthermore, obesity frequently displays synergy with exposures upon health outcomes, including hypertension. Therefore, obesity could exacerbate the effect of adverse menstrual characteristics to increase the prevalence of hypertension. Hence, here we investigated the association between menstrual abnormalities, including cycle length, regularity, bleeding duration, blood loss and dysmenorrhea, and the prevalence of hypertension (normal, elevated, Stage 1 and Stage 2) in young adult women (n = 178,205). We then examined how obesity modifies the effect of menstrual abnormalities upon the prevalence of hypertension. We report that overweight/obesity amplified the effect of the association between menstrual abnormalities and hypertension in young adults.

## Materials and methods

### Participants

Project Environmental and LifEstyle FActors iN metabolic health throughout life-course Trajectories (ELEFANT) incorporates three population-based studies: Baby (mean age 0), Young (mean age 29) and Elderly ELEFANT (mean age 69). All participants resided in Tianjin (area: 11760 km^2^), China at the time of recruitment. In this study, we utilized the Young ELEFANT cohort, using cross-sectional data from 2011–2014. The total number of participants within Young ELEFANT is 356,410 (50% women). Basic demographic and clinical characteristics were measured during routine checkups. The participants also completed questionnaires which assessed lifestyles such as smoking, alcohol consumption, psychological stress, education level, occupation, reproductive characteristics (age at menarche, menstrual cycle length, menstrual bleeding duration, dysmenorrheal and parity), history of disease, familial history of disease, medication use, and others. The protocol of this study was approved by the Institutional Review Board of the Tianjin Medical University before the experiment was started and participants gave written informed consent prior to participation in the study.

### Study design

**Fig 1:**
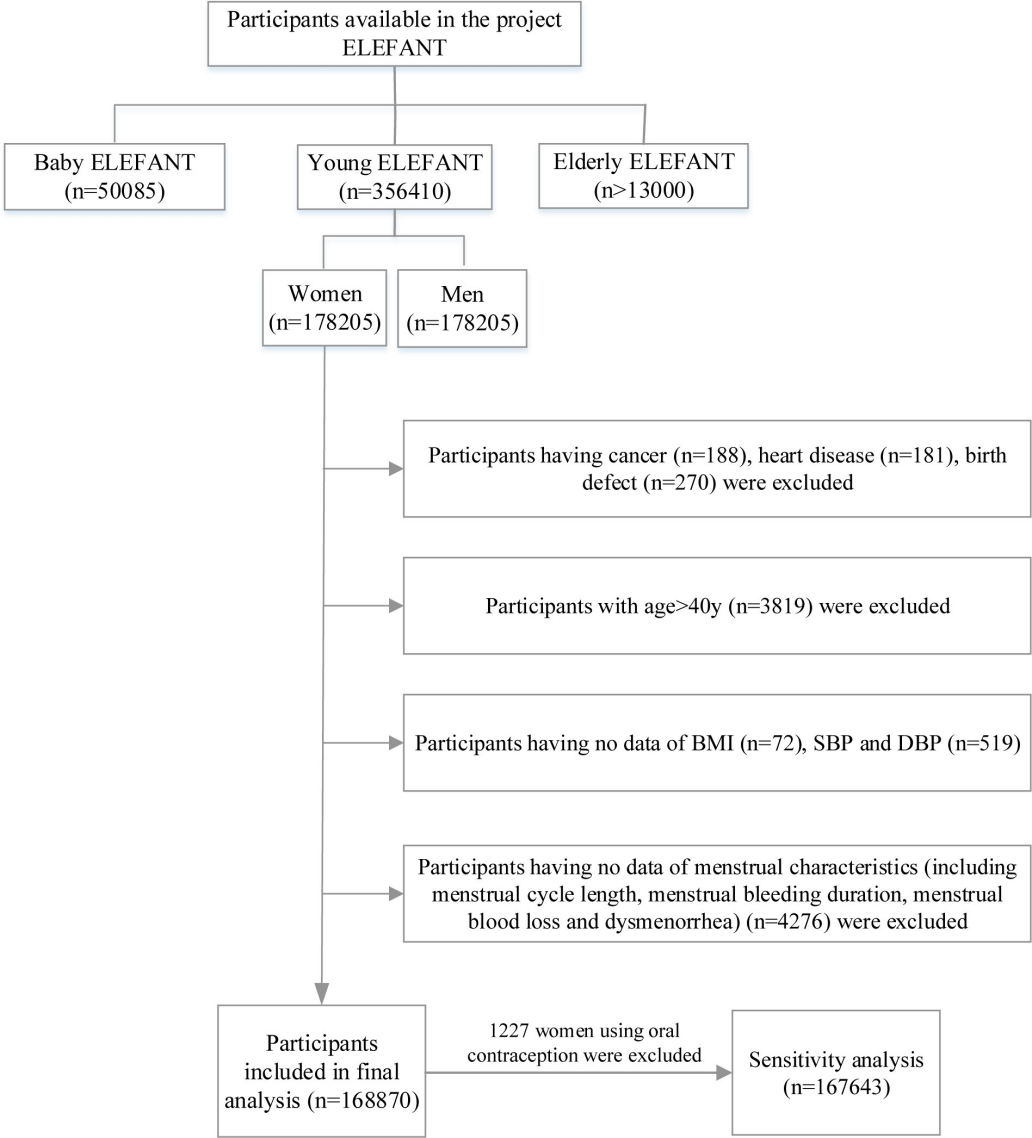
Study design and participant flow chart for the present study.

The Young ELEFANT cohort contains a total of 178,205 female participants (Fig 1). We excluded participants with a history of cancer, heart disease, birth defect and participants aged >40 years in order to improve homogeneity of the study population and avoid the instability of menstrual characteristics during pre-menopause. Participants with missing data were also excluded. Following these exclusions, 168,870 participants were included in the final analysis. Furthermore, considering that oral contraception might affect menstrual characteristics, a sensitivity analysis was performed in participants not using the oral contraceptive (*n* = 167,643).

### Assessment of hypertension

After 5 to 10 minutes of rest, individual blood pressure was measured twice in the upper left arm in a seated position using an automatic device (HBP-9021J, Omron, Japan). The blood pressure value was defined as the mean of the two measurements. Hypertension was diagnosed according to the 2017 High Blood Pressure Clinical Practice Guideline [19] and thereby categorized into normal blood pressure (systolic blood pressure (SBP)<120 mm Hg and diastolic blood pressure (DBP)<80 mm Hg), elevated (SBP 120–129 mm Hg and DBP<80 mm Hg), hypertension Stage 1 (SBP 130–139 mm Hg or DBP 80–89 mm Hg), and hypertension Stage 2 (SBP ≥140 mm Hg or DBP≥90 mm Hg).

### Assessment of menstrual characteristics

Women reported their usual menstrual cycle characteristics including cycle length, bleeding duration, blood loss, and having dysmenorrhea in the aforementioned questionnaires. The participants reported the upper and lower limit of cycle length and bleeding duration. Irregular cycles were defined as a difference of 7 days or more in cycle length between the upper and lower limits [20]. Polymenorrhea was defined with average cycle of 21 days and oligomenorrhea was having average cycle of 36 days and longer, in line with studies elsewhere [21]. Normal cycle was dichotomized into short normal (>21 and ≤29 days) and long normal (>29 and ≤35 days), as 29 days was the median in the cohort. The normal menstrual bleeding duration ranged between 3 to 7 days [21]. A menstrual pictogram was provided for all participants to evaluate their usual menstrual blood loss [5, 22], with the amount of menstrual blood loss estimated using the images and self-reported usual numbers of sanitary napkins or tampons used, which was categorized as light (<20 mL), medium (20-80mL), and heavy (>80 mL). Dysmenorrhea was defined as crampy and spasmodic pain in lower abdomen radiating to the back and thighs, which begins just before or as menstrual bleeding begins, and gradually diminishes over 1 to 3 days [23].

### Assessment of other variables

Data on age at enrollment, education, occupation, lifestyles, family history of disease, reproductive characteristics and medication usage were collected in the interview utilizing a structured questionnaire. Education was calculated by the maximum years of formal schooling and classified into three categories of ≤9, 10~15 and ≥16 years. Occupation was categorized as manual work, non-manual work or unemployment. According to their registered permanent residence, the region in which the participants lived was dichotomized into rural and urban. Cigarette smoking (current smokers vs. former or non-smokers), passive smoking (current passive smokers vs. former or non- passive smokers) and alcohol consumption (current drinkers vs. former or non-drinkers) were dichotomized. Psychological stress, including work-related, social and economic stress, was also dichotomized. Parity was dichotomized as one or fewer vs. two or more. The use of oral contraceptive pill was grouped as current, former user and non-user. Family history of hypertension was dichotomized to ‘yes’ or ‘no’. According a standard protocol, we measured anthropometric measures of each participant, and body mass index (BMI) was calculated as weight/height^2^ (kg/m^2^). According to clinical standards in China, overweight was defined as BMI between 24.0 kg/m^2^ and 28.0 kg/m^2^ and obesity was as BMI ≥ 28.0 kg/m^2^ [24].

### Statistical analysis

Characteristics between normotension and hypertension were analyzed with t tests for normal distributive continuous variables, Wilcoxon test for skewed distributive variables and Chi-square tests or Fisher test for categorical variables. Logistic regression analyses were performed to estimate the odds ratios (ORs) and 95% confidence intervals (CIs) for hypertension in relation to menstrual characteristics including menstrual cycle (reference group: short normal cycle, of 21–29 days), menstrual bleeding duration (reference group: 3–7 days), menstrual blood loss (reference group: medium) and dysmenorrhea. To understand whether potential confounders could affect the ORs, we adjusted our models for age, BMI, fasting blood glucose level (FBG), smoking status, passive smoking status, alcohol consumption, education, occupation, region, parity, oral contraceptive use, age at menarche and family history of hypertension. The associations between BMI groups were analyzed by healthy weight (18.5≤BMI < 24) or overweight and obesity (BMI ≥ 24). We performed the multiplicative interactions between menstrual characteristics and BMI groups inputting the product interaction terms in the logistic regression model adjusting the potential confounders using the likelihood ratio test. Sensitivity analyses were performed with participants not using oral contraception. All statistical analyses were performed using SAS 9.4 (SAS Institute Inc, Cary, NC, USA). A two-tailed *p* value of less than 0.05 was considered to be statistically significant.

## Results

A total of 168,870 young women were analyzed in this cross-sectional study. Among the young adult women, 6.05% had elevated blood pressure, 30.46% Stage 1 hypertension, and 3.28% Stage 2 hypertension (Table 1). The mean age of the participants was 28 in normotensive group and 30 in Stage 2 hypertension (Table 1).

**Table 1:** Basic characteristics of the study population.

Variables	Normotension	Elevated	Stage 1	Stage 2	P*
**Total,** *n* (%)	101671 (60.21%)	10220 (6.05%)	51440 (30.46%)	5539 (3.28%)	
**Age,** years (SD)	28 (4.14)	29 (4.28)	28 (4.39)	30 (4.51)	<0.0001
**BMI,** kg/m^2^ (SD)	22 (3.94)	23 (4.52)	23 (3.95)	26 (4.78)	<0.0001
**Overweight,** *n* (%)	15492 (15.24%)	2163 (21.16%)	11892 (23.12%)	1707 (30.82%)	<0.0001
**Obesity,** *n* (%)	4500 (4.43%)	851 (8.33%)	4970 (9.66%)	1526 (27.55%)	<0.0001
**SBP,** mmHg (SD)	105.91 (6.28)	121.03 (2.15)	119.68 (5.56)	137.75 (14.54)	<0.0001
**DBP,** mmHg (SD)	67.26 (5.23)	71.45 (4.13)	80.32 (2.08)	91.84 (9.11)	<0.0001
**FBG,** mmol/L(SD)	5.00 (0.95)	5.11 (1.32)	5.08 (1.23)	5.53 (1.91)	<0.0001
**Drinking,** *n* (%)	5984 (5.90%)	615 (6.03%)	2682 (5.23%)	458 (8.30%)	<0.0001
**Smoking,** *n* (%)	1183 (1.17%)	157 (1.54%)	550 (1.07%)	118 (2.14%)	<0.0001
**Passive smoking,** *n* (%)	21890 (21.57%)	2469 (24.19%)	11301 (22.01%)	1715(31.02%)	<0.0001
**Education**					
≤9 years, *n* (%)	43971 (43.32%)	4820 (47.31%)	25327 (49.31%)	2812 (51.00%)	<0.0001
10~15 years, *n* (%)	17536 (17.29%)	1546 (15.17%)	7997 (15.57%)	867 (15.72%)	
≥16 years, *n* (%)	39965 (39.40%)	3823 (37.52%)	18035 (35.12%)	1835 (33.28%)	
**Occupation**					
Manual work, *n* (%)	74328 (73.42%)	7594 (74.83%)	39972 (78.00%)	4122 (74.82%)	<0.0001
Non-manual work, *n* (%)	22404 (22.13%)	2085 (20.54%)	9214 (17.98%)	1052 (19.10%)	
Unemployment, *n* (%)	4507 (4.45%)	470 (4.63%)	2062 (4.02%)	335 (6.08%)	
**Region**					
Rural, *n* (%)	73152 (71.94%)	7427 (72.67%)	40015 (77.79%)	3927 (70.90%)	<0.0001
Urban, *n* (%)	28519 (28.06%)	2793 (27.33%)	11425 (22.21%)	1612 (29.10%)	
**Psychological stress,** *n* (%)	21876 (21.58%)	2458 (24.08%)	10436 (20.33%)	1655 (29.94%)	<0.0001
**Oral contraception use,** *n* (%)	685 (0.67%)	115 (1.13%)	368 (0.72%)	59 (1.07%)	<0.0001
**Parity**					
None, *n* (%)	54922 (54.06%)	5034 (49.29%)	26390 (51.33%)	2495 (45.07%)	<0.0001
≥1 times, *n* (%)	46666 (45.94%)	5180 (50.71%)	25024 (48.67%)	3041 (54.93%)	
**Age at menarche,** years (SD)	13.85 (1.35)	13.87 (1.40)	13.88 (1.35)	13.84 (1.62)	0.0082
**Menstrual cycle,** days (SD)	29.91 (5.24)	29.96 (7.14)	29.89 (5.84)	30.60 (7.23)	<0.0001
**Menstrual cycle**					
Regular cycle, *n* (%)	94788 (93.26%)	9664 (94.56%)	48826 (94.92%)	5081 (91.73%)	<0.0001
Irregular cycle, *n* (%)	6883 (6.74%)	556 (5.44%)	2614 (5.08%)	458 (8.27%)	
**Menstrual bleeding duration,** days (SD)	5.42 (1.21)	5.46 (1.42)	5.22 (1.21)	5.62 (1.26)	<0.0001
**Menstrual blood loss**					
<20 mL, *n* (%)	4770 (4.69%)	449 (4.39%)	2252 (4.38%)	410 (7.40%)	0.0010
20-80mL, *n* (%)	93414 (91.88%)	9330 (91.29%)	47145 (91.65%)	4770 (86.12%)	
>80mL, *n* (%)	3486 (3.43%)	441 (4.32%)	2043 (3.97%)	359 (6.48%)	
**Dysmenorrhea**, *n* (%)	42124 (41.44%)	3769 (36.88%)	16788 (32.64%)	2489 (44.94%)	
**Family history of hypertension**, *n* (%)	169 (0.17%)	33 (0.32%)	31 (0.06%)	7 (0.13%)	<0.0001

Continuous variables were expressed by means (SD) and categorical variables were expressed by number (%). BMI: body mass index; SBP: systolic blood pressure; DBP: diastolic blood pressure, T2DM: type 2 diabetes mellitus.

*Comparison between normotension, elevated blood pressure, stage 1 and stage 2 hypertension: *p*-value from ANOVA, chi-square test or fisher test.

Numbers of subjects with missing value were 306 for passive smoking, 319 for smoke, 483 for drinking, 367 for education, 725 for occupation, 410 for psychological stress, 118 for parity, and 89 for age at menarche.

**Fig 2:**
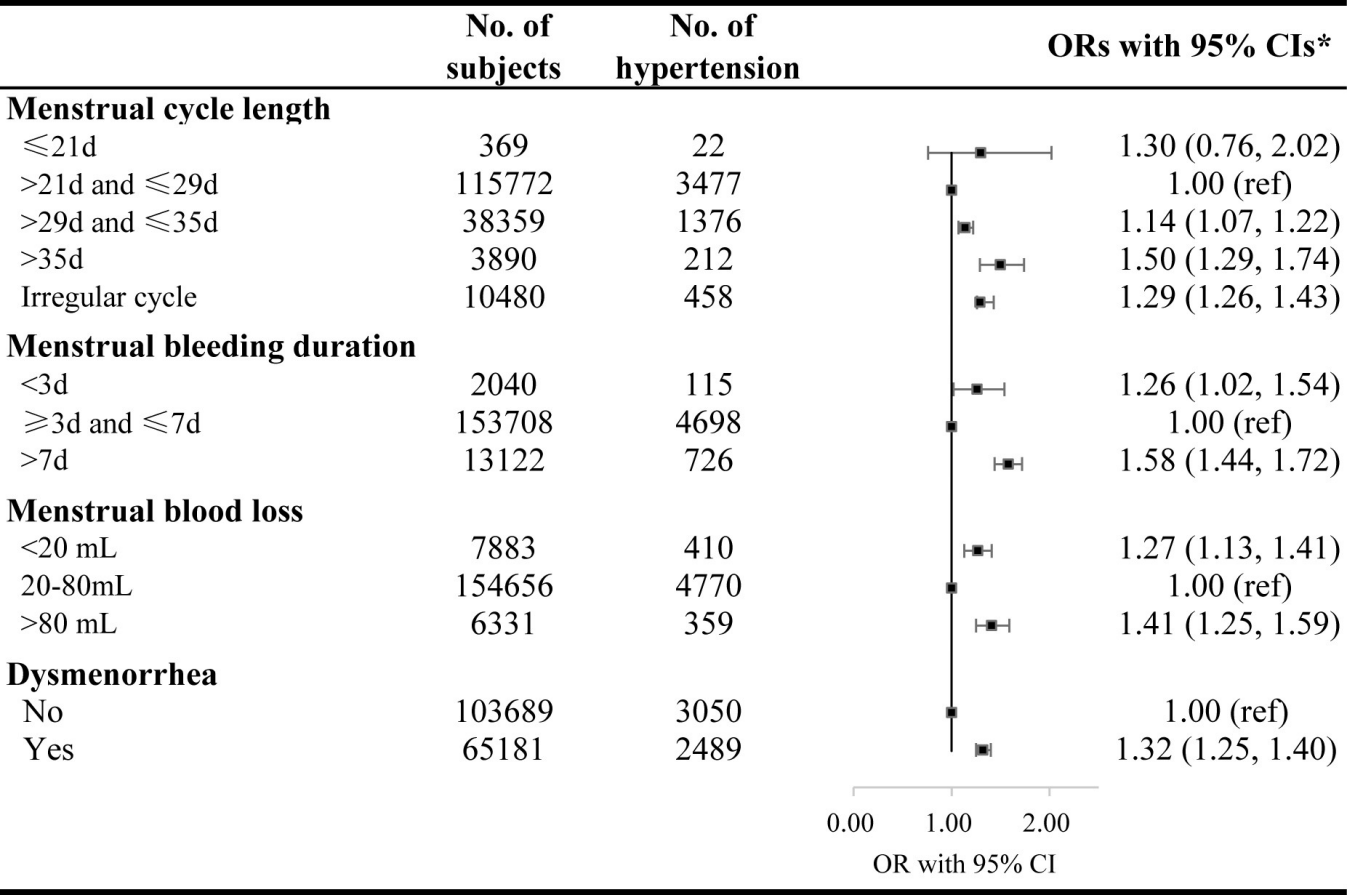
The ORs with 95% CIs for Stage 2 hypertension by menstrual abnormalities. ORs were adjusted for age at enrollment, smoking, passive smoking, drinking, FBG, education, occupation, region, psychological stress, parity, oral contraceptive use, and age at menarche.

Menstrual abnormalities including menstrual cycle length, bleeding duration, blood loss and dysmenorrhea were examined with the prevalence of hypertension. The multivariable adjusted models showed that the odds of having Stage 2 hypertension were higher in women with longer menstrual cycle length (>29 and ≤35 days: OR 1.14, 95% CI 1.07–1.22; and >35 days: OR 1.50, 95% CI 1.29–1.74) and irregular cycle (OR 1.29, 95% CI 1.26–1.43). The ORs of Stage 2 hypertension were also higher with menstrual bleeding duration of less than 3 days (1.26, 95% CI 1.02–1.54) or longer than 7 days (1.58, 95% CI 1.44–1.72). Young women with menstrual blood loss of <20 mL or >80mL had significantly higher ORs for Stage 2 hypertension (1.27, 95% CI 1.13–1.41 and 1.41, 95% CI 1.25–1.59, respectively). Young women with dysmenorrhea showed significantly higher OR for Stage 2 hypertension than women without it (1.32, 95% CI 1.25–1.40) (Fig 2). The ORs for elevated blood pressure and Stage 1 hypertension did not increase with menstrual abnormalities, except menstrual blood loss (1.20, 95% CI 1.08–1.33 for elevated and 1.11, 95% CI 1.05–1.17 for Stage 1). The model was adjusted for age at enrollment, smoking, passive smoking, alcohol consumption, BMI, FBG, education, occupation, region, psychological stress, parity, oral contraceptive use, age at menarche and family history of hypertension (Table 2).

**Table 2:** The ORs with 95% CIs for elevated, stage 1 and stage 2 hypertension by menstrual abnormalities.

			Total *n*	*n*	OR (95%CI)*	*P*_interaction_
**Elevated (*n* = 10220)**	**Menstrual cycle length**	≤21d	369	21	0.94 (0.58, 1.46)	0.8837
		>21d and ≤29d	115772	6960	1.00 (ref)	
		>29d and ≤35d	38359	2463	**1.06 (1.01, 1.12)**	0.5049
		>35d	3890	220	0.91 (0.79, 1.05)	0.6308
		Irregular cycle	10480	556	0.82 (0.75, 0.90)	0.2790
	**Menstrual bleeding duration**	<3d	2040	134	1.01 (0.84, 1.21)	0.1910
		≥3d and ≤7d	153708	9221	1.00 (ref)	
		>7d	13122	865	1.07 (0.99, 1.16)	0.8925
	**Menstrual blood loss**	<20 mL	7883	449	0.87 (0.78, 0.96)	0.9365
		20-80mL	154656	9330	1.00 (ref)	
		>80mL	6331	441	**1.20 (1.08, 1.33)**	0.9468
	**Dysmenorrhea**	No	103689	6451	1.00 (ref)	
		Yes	65181	3769	0.83 (0.79, 0.87)	0.5372
**Stage 1 (*n* = 52440)**	**Menstrual cycle length**	≤21d	369	123	1.12 (0.89, 1.40)	0.4714
		>21d and ≤29d	115772	36392	1.00 (ref)	
		>29d and ≤35d	38359	11174	0.94 (0.92, 0.97)	**0.0420**
		>35d	3890	1137	0.97 (0.90, 1.04)	**0.0206**
		Irregular cycle	10480	2614	0.74 (0.70, 0.77)	**0.0225**
	**Menstrual bleeding duration**	<3d	2040	581	0.90 (0.81, 1.00)	0.3369
		≥3d and ≤7d	153708	47278	1.00 (ref)	
		>7d	13122	3581	0.95 (0.91, 1.01)	**0.0078**
	**Menstrual blood loss**	<20 mL	7883	2252	0.94 (0.90, 1.00)	0.5471
		20-80mL	154656	47145	1.00 (ref)	
		>80mL	6331	2043	**1.11 (1.05, 1.17)**	0.1325
	**Dysmenorrhea**	No	103689	35652	1.00 (ref)	
		Yes	65181	16788	0.73 (0.71, 0.74)	0.6994
**Stage 2 (*n* = 5539)**	**Menstrual cycle length**	≤21d	369	22	1.30 (0.76, 2.02)	0.3225
		>21d and ≤29d	115772	3477	1.00 (ref)	
		>29d and ≤35d	38359	1376	**1.14 (1.07, 1.22)**	0.5974
		>35d	3890	212	**1.50 (1.29, 1.74)**	**0.0268**
		Irregular cycle	10480	458	**1.29 (1.16, 1.43)**	0.0946
	**Menstrual bleeding duration**	<3d	2040	115	**1.26 (1.02, 1.54**)	0.4944
		≥3d and ≤7d	153708	4698	1.00 (ref)	
		>7d	13122	726	**1.58 (1.44, 1.72)**	**<0.0001**
	**Menstrual blood loss**	<20 mL	7883	410	**1.27 (1.13, 1.41**)	**0.0217**
		20-80mL	154656	4770	1.00 (ref)	
		>80mL	6331	359	**1.41 (1.25, 1.59)**	0.6142
	**Dysmenorrhea**	No	103689	3050	1.00 (ref)	
		Yes	65181	2489	**1.32 (1.25, 1.40)**	0.2806

*Adjusted for age at enrollment, smoking, passive smoking, drinking, BMI, FBG, education, occupation, region, psychological stress, parity, oral contraceptive use, age at menarche, family history of hypertension.

***P***_**interaction**_ is the p value for interaction between BMI and menstrual characteristics.

**Fig 3:**
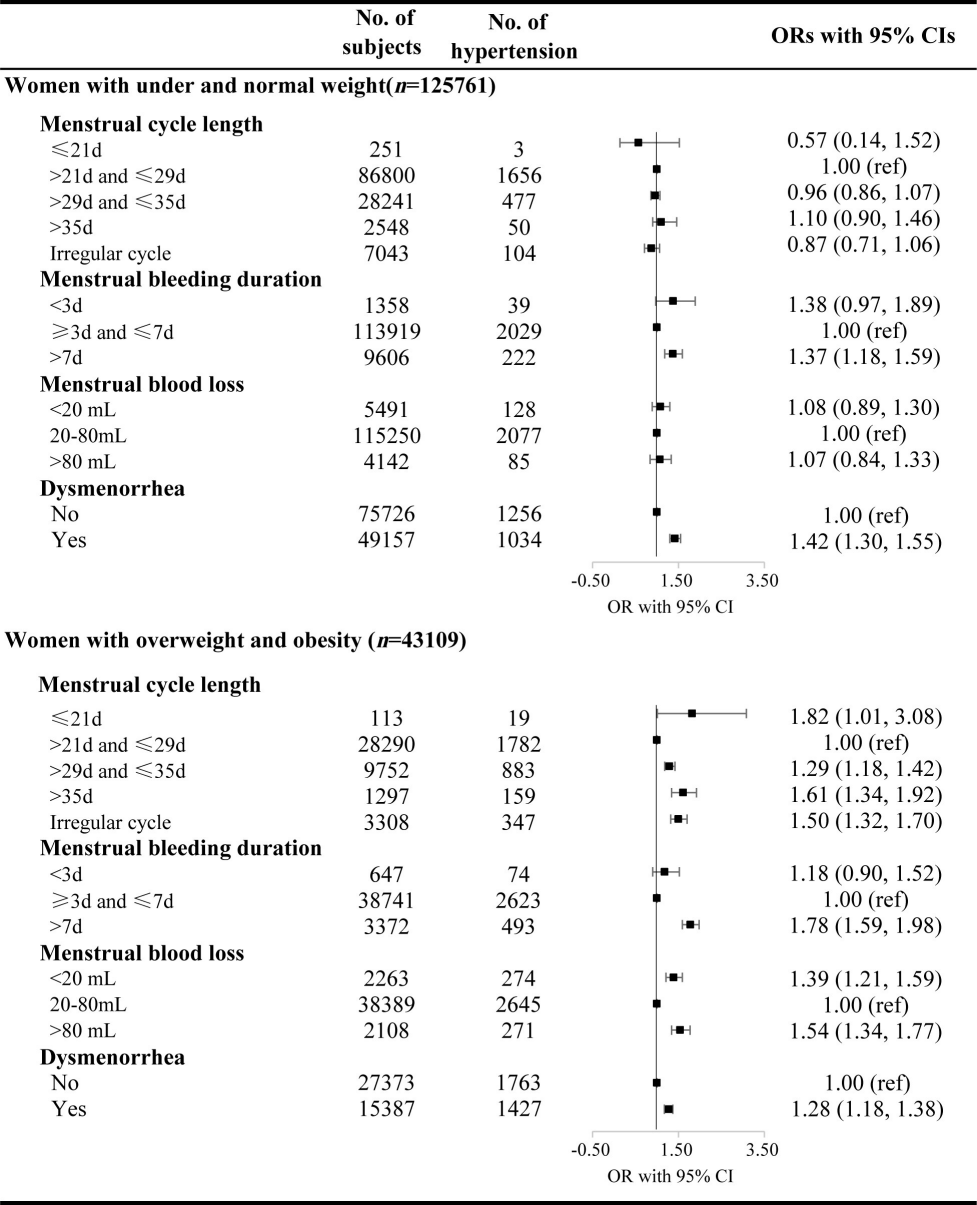
The ORs with 95% CIs for Stage 2 hypertension by menstrual abnormalities in different BMI categories. ORs were adjusted for age at enrollment, smoking, passive smoking, drinking, FBG, education, occupation, region, psychological stress, parity, oral contraceptive use, and age at menarche.

The associations between menstrual characteristics including menstrual cycle length, bleeding duration and blood loss and the prevalence of hypertension were different by BMI group (18.5≤BMI< 24, healthy weight vs BMI ≥24, overweight and obese) (Tables 3 and 4, related *P* for interaction is <0.05). Among healthy weight individuals, the ORs for elevated blood pressure were higher with menstrual cycle length of >29d and ≤35d (1.05, 95% CI 1.00–1.12), menstrual bleeding duration of > 7days (1.12, 95% CI 1.02–1.23), and menstrual blood loss of >80mL (1.25, 95% CI 1.09–1.42). The ORs for Stage 2 were higher with menstrual bleeding duration of > 7days (1.36, 95% CI 1.16–1.59) and dysmenorrhea (1.45, 95% CI 1.32–1.59) (Fig 3 and Table 3). Participants with underweight are shown in S1 Table.

**Table 3:** The ORs with 95% CIs for elevated, stage 1 and stage 2 hypertension by menstrual abnormalities among young women in who are healthy weight.

			Healthy weight (18.5≤BMI< 24, *n* = 112149)					
			Elevated (*n* = 6551)		Stage 1 (*n* = 31395)		Stage 2 (*n* = 2183)	
		Total *n*	*n*	OR (95% CI)*	*n*	OR (95% CI)*	*n*	OR (95% CI)*
**Menstrual cycle length**	≤21d	214	15	1.27 (0.71, 2.11)	75	1.32 (0.98, 1.76)	2	0.41 (0.07, 1.29)
	>21d and ≤29d	78605	4551	1.00 (ref)	22958	1.00 (ref)	1589	1.00 (ref)
	>29d and ≤35d	24882	1564	**1.05 (1.00, 1.12)**	6525	0.91 (0.88, 0.94)	446	0.95 (0.85, 1.06)
	>35d	2218	119	0.85 (0.70, 1.03)	559	0.91 (0.82, 1.00)	48	1.21 (0.82, 1.50)
	Irregular cycle	6230	302	0.75 (0.66, 0.84)	1278	0.67 (0.62, 0.71)	98	0.86 (0.70, 1.06)
**Menstrual bleeding duration**	<3d	1237	78	0.98 (0.77, 1.24)	310	0.88 (0.77, 1.01)	36	1.33 (0.93, 1.85)
	≥3d and ≤7d	102771	5917	1.00 (ref)	29095	1.00 (ref)	1943	1.00 (ref)
	>7d	8141	556	**1.12 (1.02, 1.23)**	1990	0.90 (0.85, 0.95)	204	**1.36 (1.16, 1.59)**
**Menstrual blood loss**	<20 mL	4791	274	0.88 (0.77, 1.00)	1220	0.91 (0.85, 0.98)	118	1.05 (0.86, 1.27)
	20-80mL	103670	6008	1.00 (ref)	29150	1.00 (ref)	1983	1.00 (ref)
	>80mL	3688	269	**1.25 (1.09, 1.42)**	1025	1.01 (0.94, 1.10)	82	1.08 (0.85, 1.36)
**Dysmenorrhea**	No	68791	4085	1.00 (ref)	21352	1.00 (ref)	1198	1.00 (ref)
	Yes	43358	2466	0.85 (0.80, 0.90)	10043	0.69 (0.67, 0.71)	985	**1.45 (1.32, 1.59)**

*Adjusted for age at enrollment, smoking, passive smoking, drinking, BMI, FBG, education, occupation, region, psychological stress, parity, oral contraceptive use, age at menarche, family history of hypertension.

**Table 4:** ORs with 95% CIs for elevated, stage 1 and stage 2 hypertension by menstrual abnormalities among young women who are overweight and obese.

		Overweight and obese (BMI ≥24, *n* = 43109)						
			Elevated (*n* = 10220)		Stage 1 (*n* = 52440)		Stage 2 (*n* = 5539)	
		Total *n*	*n*	OR (95% CI)*	*n*	OR (95% CI)*	*n*	OR (95% CI)*
**Menstrual cycle length**	≤21d	114	5	0.62 (0.21, 1.41)	40	0.95 (0.62, 1.44)	19	1.76 (0.98, 2.98)
	>21d and ≤29d	28465	1988	1.00 (ref)	11243	1.00 (ref)	1804	1.00 (ref)
	>29d and ≤35d	9862	714	1.05 (0.96, 1.16)	3863	1.04 (0.99, 1.09)	894	**1.28 (1.17, 1.40)**
	>35d	1315	86	0.97 (0.76, 1.23)	519	1.11 (0.98, 1.26)	162	**1.61 (1.34, 1.93)**
	Irregular cycle	3353	221	0.93 (0.80, 1.08)	1197	0.91 (0.84, 0.98)	354	**1.50 (1.32, 1.70)**
**Menstrual bleeding duration**	<3d	658	47	1.00 (0.72, 1.36)	241	0.92 (0.77, 1.09)	76	1.19 (0.91, 1.53)
	≥3d and ≤7d	39038	2729	1.00 (ref)	15325	1.00 (ref)	2656	1.00 (ref)
	>7d	3413	238	1.06 (0.91, 1.23)	1296	**1.08 (1.00, 1.16)**	501	**1.78 (1.59, 1.99)**
**Menstrual blood loss**	<20 mL	2294	145	0.88 (0.73, 1.05)	868	1.01 (0.92, 1.11)	281	**1.40 (1.22, 1.61)**
	20-80mL	38682	2721	1.00 (ref)	15093	1.00 (ref)	2678	1.00 (ref)
	>80 mL	2133	148	1.15 (0.95, 1.37)	901	**1.27 (1.15, 1.40)**	274	**1.53 (1.33, 1.76)**
**Dysmenorrhea**	No	27545	1975	1.00 (ref)	11328	1.00 (ref)	1787	1.00 (ref)
	Yes	15564	1039	0.80 (0.74, 0.88)	5534	0.78 (0.75, 0.82)	1446	**1.27 (1.17, 1.37)**

*Adjusted for age at enrollment, smoking, passive smoking, drinking, BMI, FBG, education, occupation, region, psychological stress, parity, oral contraceptive use, age at menarche, and family history of hypertension.

However, among overweight and obese women, the ORs of Stage 2 hypertension were significantly higher in participants by most of the menstrual abnormalities, including menstrual cycle length of 29-35days and >35days, irregular cycle, menstrual bleeding duration of >7days, menstrual blood loss of >80 mL and dysmenorrhea (Fig 3 and Table 4). In women with menstrual bleeding duration of >7days or menstrual blood loss of >80 mL, significantly higher ORs were present for Stage 1 hypertension (Table 4).

In the sensitivity analyses after excluding the women who reported using the oral contraceptive pill, we found similar results as when they were included (S2 Table), including when categorized by BMI (S3 Table).

## Discussion

The present study assessed the relationship between menstrual abnormalities with hypertension by blood pressure and the role of obesity in this association in young Chinese women. We found that longer menstrual cycle (>29 days), irregular cycle, light (<20mL) and heavy (>80mL) menstrual blood loss were associated with Stage 2 hypertension among overweight and obese women, but not in normal weight women (BMI ≥24). Longer duration of menstrual bleeding (>7days) and dysmenorrhea were associated with Stage 2 hypertension irrespective of BMI. Our results suggest that BMI may modify the association between menstrual abnormalities and hypertension in young women.

There have been conflicting results from studies investigating the association between menstrual cycle characteristics and hypertension. Irregular menstrual cycles and oligomenorrhea have been reported to be potential markers of CVD risk, including coronary heart disease, in a cohort study of European women, and were also associated with arterial hypertension in a retrospective case-control study of 414 postmenopausal Brazilian women [25, 26]. Longer menstrual cycle length has also been reported to be associated with CVD risk factors such as blood lipid levels and DBP [27]. However, a retrospective case-control study of Chinese women reported no association between menstrual cycle length and hypertension among postmenopausal women [28]. We speculate that these conflicting results may in part be the product of the retrospective studies being conducted in postmenopausal women, thereby potentially being affected by menstrual irregularities during the pre-menopause. Furthermore, obesity has been shown to be significantly associated with having an irregular menstrual cycle. To the best of our knowledge, our study is the largest to examine the association between menstrual cycle characteristics and the prevalence of hypertension, and the first to examine the modifying effect of obesity. We report that oligomenorrhea (36 days and longer), normal prolonged menstrual cycle (29–35 days) and irregular menstrual cycle were each associated with the prevalence of Stage 2 hypertension in young obese women but not in healthy weight women.

The underlying mechanism explaining the relationship between menstrual cycle length and hypertension in obese women is unclear. Lower estrogen levels can lead to a longer or irregular menstrual cycle in women [7, 17]. In addition, these can be the product of inadequate follicular development leading to prolonging of the follicular phase of the cycle, which in itself is often associated with lower average estrogen concentrations [7, 17, 29, 30]. It is known that estradiol (E2) can execute vasodilator function by augmentation of the beta-adrenergic receptor, and reducing oxidative stress [12, 13]. Thus, we speculate that decreased estrogen levels may explain the mechanism underlying the association between irregular or prolonged menstrual cycle length and hypertension. In addition, obese women have much greater amplitudes of changes in sex hormone levels across the menstrual cycle to maintain hormonal homeostasis compared to normal weight women [31]. Adiposity itself can contribute to endothelial or vasodilator dysfunction by production of reactive oxygen species (ROS) and inflammation mediators, similar to lower estrogen levels [32]. Indeed, lower estrogen levels together with increased BMI could bring out apparent impairment of vascular function such as hypertension. This may explain why the association between menstrual cycle and hypertension was only observed among obesity or overweight women.

There have been few previous reports on menstrual bleeding patterns, such as duration and blood loss, with hypertension and CVD. Just as with cycle characteristics, we observed light menstrual blood loss to be associated with increased odds of hypertension only among young women who are overweight or obese, and longer menstrual bleeding duration was also associated with prevalence of hypertension among overweight and obese women. During a normal menstrual period, estrogen levels reduce a few days before the endometrium thickens [33]. If the levels of estrogen are reduced abnormally, this slows the endometrial repair period, contributing to longer bleeding duration and relatively lighter blood loss [34]. Moreover, long bleeding durations are associated with anovulatory cycles which relate to significant reduction in estradiol level [35]. Therefore, it could be that it is changes in estrogen levels that determine the increased prevalence of hypertension among young obese women with longer bleeding duration and lighter blood loss.

Interestingly, dysmenorrhea was shown to be related with increased prevalence of hypertension in both obese and non-obese women in our study. A previous case-control study in Italy reported that the occurrence of pregnancy-related hypertensive disorders including gestational hypertension and preeclampsia is influenced by dysmenorrhea [36]. Compared with unmenorrheic women, dysmenorrheic women have higher levels of prostaglandins (PGs) in both endometrial biopsies and circulation, predominantly PGF_2a_ and PGE_2_ [6], and PGF2_a_ is known to elevate blood pressure via vasoconstrictor function [35]. The mechanism underlying this association requires further study.

Several limitations need to be acknowledged. Firstly, information on menstrual cycle were self-reported by the participants, and therefore recall bias can be present. Although, questionnaire data has been reported to be more accurate among younger participants [25, 37], response bias has been a key issue in many studies using self-assessed questionnaire data. Recalibration of response bias has been suggested, however this can cause ‘response-shift bias’ or other types of response bias [38]. Furthermore, this also requires data from multiple measures and observations [38], which was not available for our analysis. Secondly, due to lack of long-term follow-up data, we cannot infer causality of hypertension with menstrual characteristics. Thirdly, non-steroidal anti-inflammatory drugs (NSAIDs) can be used to alleviate the symptoms of dysmenorrhea, and their chronic usage has been linked with hypertension [39]. However, there is currently no data on NSAID usage within this cohort, and therefore we have been unable to exclude their confounding effect. Indeed, cross-sectional studies such as ours are prone to confounding due to the wide range of factors that may be associated with the outcome. We therefore sought to counter this by adjusting our model for a number of factors known to be associated with hypertension and for which data was available within the cohort (e.g. age, BMI, smoking, fasting blood glucose levels, familial history of the disease), but we cannot exclude the possibility of residual confounding. Finally, the absence of a similarly-sized population study precluded the validation of our findings in an independent cohort. Nonetheless, our study contains several strengths. Firstly, our study has a large sample size and extensive data on menstrual characteristics [26], which we leveraged to comprehensively analyze the relationship between menstrual characteristics and hypertension. Secondly, the age range of our participants was narrow, and the relatively young age of the participants meant we were able to avoid the instability of menstrual characteristics during pre-menopause. Thirdly, we were able to control for a large number of possible cofounders, including the use of oral contraceptive.

## Conclusions

In conclusion, using a large cross-sectional study based in China, we report that menstrual abnormities, including menstrual cycle length, blood loss, bleeding duration and dysmenorrhea were associated with Stage 2 hypertension, and that this association interacts with overweight and obesity. Further investigation is required to confirm our findings in independent cohorts and elucidate the biological mechanism(s) behind these associations through laboratory-based experiments.

## Supporting information

S1 Table The ORs with 95% CIs for elevated, stage 1 and stage 2 hypertension by menstrual abnormalities among young women in who are underweight. *Adjusted for age at enrollment, smoking, passive smoking, drinking, BMI, FBG, education, occupation, region, psychological stress, parity, oral contraceptive use, age at menarche, family history of hypertension.
(DOCX)

S2 Table The odds ratios (ORs) with 95% confidence intervals (95% CIs) for stage 2 hypertension by menstrual characteristics only women without using oral contraceptive. *Adjusted for age at enrollment, smoking, passive smoking, drinking, BMI, FBG, education, occupation, region, psychological stress, parity, age at menarche, family history of hypertension.
(DOCX)

S3 Table The odds ratios (ORs) with 95% confidence intervals (95% CIs) for stage 2 hypertension by menstrual abnormalities in different BMI levels among young women without using oral contraceptive. *Adjusted for age at enrollment, smoking, passive smoking, drinking, BMI, FBG, education, occupation, region, psychological stress, parity, age at menarche, family history of hypertension.
***P***_**interaction**_ is *p* value for interaction between BMI and menstrual characteristics.
(DOCX)

## Abbreviations

CVD: Cardiovascular disease
SBP: systolic blood pressure
DBP: diastolic blood pressure
Cis: confidence intervals
PMS: premenstrual syndrome
ELEFANT: Environmental and LifEstyle FActors iN metabolic health throughout life-course Trajectories
BP: Blood pressure
ORs: odds ratios
