# Supplementary material for: Obesity as an effect modifier of the association between menstrual abnormalities and hypertension in young adult women: Results from Project ELEFANT
Source: PLoS One. 2018 Nov 28;13(11):e0207929. doi: 10.1371/journal.pone.0207929 (PMC6261602; doi:10.1371/journal.pone.0207929)
Supplement: S1 Table — *Adjusted for age at enrollment, smoking, passive smoking, drinking, BMI, FBG, education, occupation, region, psychological stress, parity, oral contraceptive use, age at menarche, family history of hypertension. (DOCX) [file pone.0207929.s001.docx]

**S1 Table. The ORs with 95% CIs for Elevated, Stage 1 and Stage 2 hypertension by menstrual abnormalities among young women in who are underweight.**

|  |  |  | **Underweight (BMI < 18.5, *n*=13612)** | | | | | |
| --- | --- | --- | --- | --- | --- | --- | --- | --- |
|  |  |  | **Elevated (*n*=655)** | | **Stage 1 (*n*=3183)** | | **Stage 2 (*n*=123)** | |
|  |  | **Total *n*** | ***n*** | **OR (95% CI)*** | ***n*** | **OR (95% CI)*** | ***n*** | **OR (95% CI)*** |
| **Menstrual cycle length** | ≤21d | 41 | 1 | 0.45 (0.03, 2.13) | 8 | 0.70 (0.28 ,1.50) | 1 | 2.45 (0.14, 12.12) |
|  | >21d and ≤29d | 8702 | 421 | 1.00 (ref) | 2191 | 1.00 (ref) | 78 | 1.00 (ref) |
|  | >29d and ≤35d | 3615 | 185 | 1.07 (0.89, 1.29) | 786 | 0.89 (0.81, 0.99) | 36 | 1.14(0.74, 1.71) |
|  | >35d | 357 | 15 | 0.87 (0.49, 1.43) | 59 | 0.70 (0.52, 0.92) | 2 | 0.61(0.10, 1.98) |
|  | Irregular cycle | 897 | 33 | 0.71 (0.48, 1.02) | 139 | 0.58 (0.48, 0.70) | 6 | 0.78(0.30, 1.67) |
| **Menstrual bleeding duration** | <3d | 145 | 9 | 1.16 (0.52, 2.27) | 30 | 0.89 (0.57, 1.34) | 3 | 1.74 (0.39, 5.06) |
|  | ≥3d and ≤7d | 11899 | 575 | 1.00 (ref) | 2858 | 1.00 (ref) | 99 | 1.00 (ref) |
|  | >7d | 1568 | 71 | 0.93 (0.71, 1.21) | 295 | 0.87 (0.75, 1.00) | 21 | 1.56 (0.91, 2.55) |
| **Menstrual blood loss** | <20 mL | 798 | 30 | 0.72 (0.48, 1.05) | 164 | 0.94 (0.78, 1.12) | 11 | 1.37 (0.68, 2.51) |
|  | 20-80mL | 12304 | 601 | 1.00 (ref) | 2902 | 1.00 (ref) | 109 | 1.00 (ref) |
|  | >80mL | 510 | 24 | 0.99 (0.63, 1.48) | 117 | 1.04 (0.84, 1.29) | 3 | 0.65 (0.16, 1.76) |
| **Dysmenorrhea** | No | 7353 | 391 | 1.00 (ref) | 1972 | 1.00 (ref) | 65 | 1.00 (ref) |
|  | Yes | 6259 | 264 | 0.75 (0.63, 0.89) | 1211 | 0.72 (0.66, 0.79) | 58 | 1.01 (0.69, 1.49) |

*Adjusted for age at enrollment, smoking, passive smoking, drinking, BMI, FBG, education, occupation, region, psychological stress, parity, oral contraceptive use, age at menarche, family history of hypertension.
