# Supplementary material for: Obesity as an effect modifier of the association between menstrual abnormalities and hypertension in young adult women: Results from Project ELEFANT
Source: PLoS One. 2018 Nov 28;13(11):e0207929. doi: 10.1371/journal.pone.0207929 (PMC6261602; doi:10.1371/journal.pone.0207929)
Supplement: S2 Table — *Adjusted for age at enrollment, smoking, passive smoking, drinking, BMI, FBG, education, occupation, region, psychological stress, parity, age at menarche, family history of hypertension. (DOCX) [file pone.0207929.s002.docx]

**S2 Table. The odds ratios (ORs) with 95% confidence intervals (95% CIs) for Stage 2 hypertension by menstrual characteristics only women without using oral contraceptive.**

|  |  | **No. of**  **subjects** | **Stage 2** | | |
| --- | --- | --- | --- | --- | --- |
|  |  |  | **No.** | **Crude OR** | **Adjusted OR*** |
|  |  | |  |  |  |
| **Menstrual cycle length** | ≤21d | 364 | 22 | **2.09 (1.32, 3.14)** | 1.31 (0.78, 2.07) |
|  | >21d and ≤29d | 115090 | 3438 | 1.00 (ref) | 1.00 (ref) |
|  | >29d and ≤35d | 37993 | 1360 | **1.21 (1.13, 1.29)** | **1.15 (1.07, 1.23)** |
|  | >35d | 3845 | 209 | **1.87 (1.61, 2.15)** | **1.50 (1.29, 1.74)** |
|  | Irregular cycle | 10351 | 451 | **1.48 (1.34, 1.63)** | **1.29 (1.16, 1.43)** |
| **Menstrual bleeding duration** | <3d | 2005 | 113 | **1.90 (1.56, .2.29)** | **1.26 (1.02, 1.54)** |
|  | ≥3d and ≤7d | 152660 | 4652 | 1.00 (ref) | 1.00 (ref) |
|  | >7d | 12978 | 715 | **1.86 (1.71, 2.01)** | **1.57 (1.44, 1.72)** |
| **Menstrual blood loss** | <20 mL | 7754 | 129 | **1.72 (1.55, 1.91)** | **1.26 (1.13, 1.41)** |
|  | 20-80mL | 153639 | 1533 | 1.00 (ref) | 1.00 (ref) |
|  | >80 mL | 356 | 94 | **1.91 (1.70, 2.13)** | **1.42 (1.26, 1.60)** |
| **Dysmenorrhea** | No | 103099 | 3019 | 1.00 (ref) | 1.00 (ref) |
|  | Yes | 64544 | 2461 | **1.31 (1.25, 1.39)** | **1.33 (1.25, 1.41)** |

*Adjusted for age at enrollment, smoking, passive smoking, drinking, BMI, FBG, education, occupation, region, psychological stress, parity, age at menarche, family history of hypertension.
