# Supplementary material for: Obesity as an effect modifier of the association between menstrual abnormalities and hypertension in young adult women: Results from Project ELEFANT
Source: PLoS One. 2018 Nov 28;13(11):e0207929. doi: 10.1371/journal.pone.0207929 (PMC6261602; doi:10.1371/journal.pone.0207929)
Supplement: S3 Table — *Adjusted for age at enrollment, smoking, passive smoking, drinking, BMI, FBG, education, occupation, region, psychological stress, parity, age at menarche, family history of hypertension. Pinteraction is p value for interaction between BMI and menstrual characteristics. (DOCX) [file pone.0207929.s003.docx]

**S3 Table. The odds ratios (ORs) with 95% confidence intervals (95% CIs) for stage 2 hypertension by menstrual abnormalities in different BMI levels among young women without using oral contraceptive.**

|  |  | **BMI < 24 kg/m^2^** | | | **BMI ≥24 kg/m^2^** | | | ***P_interaction_*** |
| --- | --- | --- | --- | --- | --- | --- | --- | --- |
|  |  | **No. of**  **subjects** | **No. of Stage 2** | **Adjusted OR*** | **No. of**  **subjects** | **No. of Stage 2** | **Adjusted OR*** |  |
| **Menstrual cycle length** | ≤21d | 251 | 3 | 0.57 (0.14, 1.52) | 113 | 19 | **1.82 (1.01, 3.08)** | 0.3171 |
|  | >21d and ≤29d | 86800 | 1656 | 1.00 (ref) | 28290 | 1782 | 1.00 (ref) |  |
|  | >29d and ≤35d | 28241 | 477 | 0.96 (0.86, 1.07) | 9752 | 883 | **1.29 (1.18, 1.42)** | 0.5528 |
|  | >35d | 2548 | 50 | 1.10 (0.90, 1.46) | 1297 | 159 | **1.61 (1.34, 1.92)** | **0.0406** |
|  | Irregular cycle | 7043 | 104 | 0.87 (0.71, 1.06) | 3308 | 347 | **1.50 (1.32, 1.70)** | 0.0866 |
| **Menstrual bleeding duration** | <3d | 1358 | 39 | 1.38 (0.97, 1.89) | 647 | 74 | 1.18 (0.90, 1.52) | 0.4387 |
|  | ≥3d and ≤7d | 113919 | 2029 | 1.00 (ref) | 38741 | 2623 | 1.00 (ref) |  |
|  | >7d | 9606 | 222 | 1.37 (1.18, 1.59) | 3372 | 493 | **1.78 (1.59, 1.98)** | **<0.0001** |
| **Menstrual blood loss** | <20 mL | 5491 | 128 | 1.08 (0.89, 1.30) | 2263 | 274 | **1.39 (1.21, 1.59)** | **0.0323** |
|  | 20-80mL | 115250 | 2077 | 1.00 (ref) | 38389 | 2645 | 1.00 (ref) |  |
|  | >80 mL | 4142 | 85 | 1.07 (0.84, 1.33) | 2108 | 271 | **1.54 (1.34, 1.77)** | 0.6555 |
| **Dysmenorrhea** | No | 75726 | 1256 | 1.00 (ref) | 27373 | 1763 | 1.00 (ref) |  |
|  | Yes | 49157 | 1034 | 1.42 (1.30, 1.55) | 15387 | 1427 | **1.28 (1.18, 1.38)** | 0.2698 |

*Adjusted for age at enrollment, smoking, passive smoking, drinking, BMI, FBG, education, occupation, region, psychological stress, parity, age at menarche, family history of hypertension.

***P*_interaction_** is *p* value for interaction between BMI and menstrual characteristics.
